# RETRACTION: Alteration of Basilar Artery Rho‐Kinase and Soluble Guanylyl Cyclase Protein Expression in a Rat Model of Cerebral Vasospasm following Subarachnoid Hemorrhage

**DOI:** 10.1155/bmri/9782469

**Published:** 2026-03-13

**Authors:** BioMed Research International

RETRACTION: C.‐J. Wang, P.‐Y. Lee, B.‐N. Wu, S.‐C. Wu, J.‐K. Loh, H.‐P. Tsai, C.‐L. Chung, N. F. Kassell, and A.‐L. Kwan, “Alteration of Basilar Artery Rho‐Kinase and Soluble Guanylyl Cyclase Protein Expression in a Rat Model of Cerebral Vasospasm following Subarachnoid Hemorrhage,” *BioMed Research International* 2014, no. 1 (2014): 1–8, https://doi.org/10.1155/2014/531508

The above article, published online on 1 June 2014 in Wiley Online Library (wileyonlinelibrary.com), has been retracted by agreement between [the authors, if applicable]; the journal Editor‐in‐Chief and John Wiley & Sons Ltd. The retraction has been agreed due to image concerns raised on PubPeer [1], specifically:

Figure 1b is identical to Figure 1c in [2]. Figure 1b reports to show the cross section of an artery from subarachnoid hemorrhage (SAH) rats, whilst Figure 1c in [2] reports to show the cross section of an artery from SAH rats treated with glycyrrhizin.

The authors were contacted for clarification but were unresponsive. Consequently, the editorial board no longer has confidence in the results and conclusions of this article.
